# Applications of Alternative Nucleases in the Age of CRISPR/Cas9

**DOI:** 10.3390/ijms18122565

**Published:** 2017-11-29

**Authors:** Tuhin K. Guha, David R. Edgell

**Affiliations:** Department of Biochemistry, Schulich School of Medicine and Dentistry, University of Western Ontario, London, ON N6A 5C1, Canada; tguha3@uwo.ca

**Keywords:** monomeric nuclease, dimeric nuclease, GIY-YIG nuclease domain, FokI, CRISPR/Cas9, ZFN, TALEN

## Abstract

Breakthroughs in the development of programmable site-specific nucleases, including zinc-finger nucleases (ZFNs), transcription activator-like effector nucleases (TALENs), meganucleases (MNs), and most recently, the clustered regularly interspaced short palindromic repeats (CRISPR) associated proteins (including Cas9) have greatly enabled and accelerated genome editing. By targeting double-strand breaks to user-defined locations, the rates of DNA repair events are greatly enhanced relative to un-catalyzed events at the same sites. However, the underlying biology of each genome-editing nuclease influences the targeting potential, the spectrum of off-target cleavages, the ease-of-use, and the types of recombination events at targeted double-strand breaks. No single genome-editing nuclease is optimized for all possible applications. Here, we focus on the diversity of nuclease domains available for genome editing, highlighting biochemical properties and the potential applications that are best suited to each domain.

## 1. Introduction

By now, almost every scientist in molecular biosciences should be familiar with CRISPR/Cas9 [1,2,3]. A component of the type II CRISPR system that constitutes the innate immune system of bacteria, the Cas9 (CRISPR-associated) protein has caused a paradigm shift in the field of genome editing due to its ease-of-use. Programming Cas9 to cleave a desired sequence is a simple matter of changing the sequence of the Cas9-associated guide RNA to be complementary to the target site [4]. The ease of programming Cas9 targeting contrasts with the more intensive protein engineering that is required for other reagents (zinc finger nucleases (ZFNs), meganucleases, transcription activator-like effector nucleases (TALENs)) [5,6,7,8,9]. The only caveat for Cas9 targeting is that an NGG protospacer-associated motif (PAM) nucleotide sequence lie immediately downstream of the target site (NGG is the PAM for the commonly used *Streptococcus pyogenes* SpCas9; other Cas9 orthologs have different PAM requirements) [10]. Cas9, along with proteins from type III CRISPR systems [11,12], have been used for a myriad of genome-editing applications in a diverse range of organisms, and are now entering the realm of therapeutic applications in humans [13]. It seems relevant, therefore, to ask the question is there a need for any other type of genome-editing reagent than Cas9? Here, we address this question by discussing the diversity of genome-editing architectures and nuclease domains, and how the different activities contribute towards specificity, targeting range, and the biasing of editing outcomes at nuclease-induced double-strand breaks (DSBs).

## 2. Genome-Editing Reagents Based on Nuclease Domains That Dimerize

### 2.1. ZFNs: Zinc-Finger Nucleases

Arguably, the first demonstration of a chimeric nuclease with genome-editing potential resulted from the fusion of the nuclease domain from the Type IIS restriction enzyme FokI to arrays of three zinc fingers where each individual zinc finger minimally recognized three bases [14]. This modular architecture mimicked that of the native FokI enzyme, a Type IIS restriction enzyme with N-terminal DNA binding and C-terminal nuclease domains that binds an asymmetric site, cleaving at a distance (S, for “shifted cleavage”), with no nucleotide preference at the cut sites (Table 1) [15]. Early versions of chimeric FokI-zinc finger fusions were tested on sites that mimicked the native FokI site, but the discovery that the nuclease domains from two FokI monomers dimerized to cleave DNA necessitated a change in the design of chimeric zinc finger nucleases [14,16]. Specifically, two FokI-zinc finger fusions, with each zinc finger targeting a different DNA sequence in a head-to-head orientation and appropriately spaced from each other to facilitate of dimerization of the FokI nuclease domains, needed to be constructed to introduce a DSB in the sequence between the two zinc finger binding sites. The potential of the zinc finger nucleases (ZFNs), thus, lied in the ability to design zinc finger domains to bind a wide range of sequences, and the non-specific (but dimeric) cleavage requirement of the FokI nuclease domain [17,18].

### 2.2. TALENs: Transcription Activator-Like Effector Nucleases

Specificity of the dimeric FokI architecture was vastly improved by the use of an alternative DNA-binding module derived from the transcription activator-like effectors (TALEs) found in the plant pathogens *Xanthomonas* and related bacteria [19,20]. TALEs possess a unique repeating structure consisting of a 33–35 amino acids where two residues of the repeat (called the repeat variable diresidue, or RVD) directly readout one DNA base [21,22]. Thus, the linear arrangement of RVDs in a TALE domain defines a one-to-one correspondence with the DNA target site. Fusion of the FokI nuclease domain to TALEs (creating TALE nucleases or TALENs) generated a genome-editing reagent with a greater DNA targeting range than ZFNs [23,24,25], and with greater specificity because TALEs could be assembled with 15–25 RVDs that would theoretically specify a unique sequence in a complex genome. Most naturally occurring TALE domains require a T at the 5′ end of the binding site (the 0 position) [19,20]. Subsequent directed evolution experiments generated TALE variants with specificity for different bases this position [26,27], thus further expanding targeting range. Genome-wide profiling studies revealed that TALENs had few off-target cleavages [28]. However, the repeated nature of the RVDs, coupled with the large coding size of TALENs (~5 kb each), posed difficulties for assembly and delivery of dimeric TALENs in size-constrained vectors, particularly in mammalian systems [29]. A related architecture, derived from the DNA-binding domain of BurrH protein (BuD) from *Burkholderia rhizoxinica*, also uses a helix-loop-helix motif similar to that found in TALE domains to read-out DNA, enabling the BuD-derived nucleases (BuDNs) [30]. 

### 2.3. Other Dimeric Nucleases

Variations on the dimeric architecture utilized a different nuclease domain in place of FokI (Table 1). A higher-specificity dimeric ZF nuclease was created by fusing the Type IIP restriction enzyme PvuII to ZFs to create ZF-PvuII nucleases [31]. Increased specificity of the ZF-PvuII nucleases relative to FokI ZFNs came from the defined cleavage requirement of PvuII (5′-CAGCTG-3′) versus the non-specific requirements of FokI, but with a reduction in the targeting potential given the predicted occurrence of PvuII sites in complex genomes (~1 in 4096 base). Interestingly, a single-chain variant of PvuII (i.e., a monomeric nuclease) was also constructed [31,32]. However, extensive non-specific cleavage by the single-chain variant precluded use in cellular systems. Increases in specificity were also observed by the replacement of the ZF binding module with a catalytically inactive I-SceI meganuclease (LAGLIDADG homing endonuclease) that has an 18-bp target site [33]. Use of inactive meganucleases as DNA targeting domains represented an increase in binding specificity that came at the cost of ease-of-use, as re-programming meganuclease binding requires extensive engineering of the protein-DNA interface. A more programmable dimeric FokI nuclease was created by fusing FokI to the Cas9 protein (Cas9-FokI) where targeting derives from the Cas9-associated sgRNA [34,35,36]. 

## 3. Monomeric Nucleases

A monomeric nuclease is a single polypeptide encoding both DNA cleavage and binding activities that does not require a higher order oligomerization for activity. One significant advantage of a monomeric over dimeric nuclease is that it reduces the engineering complexity of targeting a given sequence because only a single fusion (a monomer) need be constructed. In contrast, for dimeric genome-editing reagents, including those that are based on the FokI nuclease domains, two monomers targeting different sequences must be designed to appropriately position the nuclease domains for cleavage. An important consideration in the development of a monomeric nuclease is that it retains specificity that is appropriate for use in complex genomes. Monomeric nucleases can be generally grouped into (i) multi-domain chimeric proteins consisting of unrelated nuclease and DNA-binding domains, and (ii) single polypeptides with distinct active sites and nucleic acid binding surfaces (Table 1). Examples of monomeric nucleases will be briefly discussed. 

### 3.1. Monomeric Nucleases with Unrelated Binding and Cleavage Domains

#### 3.1.1. Single-Chain Variants of ZFNs and TALENs

Multi-domain enzymes, including FokI and other Type IIS enzymes that function as monomers, are excellent candidates for sources of nuclease domains for chimeric nucleases. Initial efforts to generate monomeric (single chain) versions of existing dimeric nucleases based on FokI and the type IIP enzyme PvuII met with variable success [31,32,37]. In another instance, single-chain ZFNs were constructed by conjugating two FokI nuclease domains, which were connected by a flexible linker, to a ZF with an N-terminal mitochondrial targeting sequence for the selective elimination of mutated mitochondrial DNA. Unlike heterodimeric ZFNs that recognizes 24 bp (12 bp by each monomer), this single-chain ZFN only recognizes 12 bp of the DNA target [38]. This lower specificity may be sufficient for targeting sites in human mitochondrial genome (~16.5 kb), but is insufficient for targeting in complex nuclear genomes.

#### 3.1.2. GIY-YIG-Derived Nucleases

These genome-editing reagents are derived from the nuclease and linker domains from the I-TevI homing endonuclease, a member of the GIY-YIG family that is named after conserved amino acids in the nuclease domain. GIY-YIG genome-editing nucleases are fusions to the N-terminus of ZFs (ZFEs), TALEs (Tev-mTALENs), meganucleases (MegaTevs), and Cas9 (TevCas9) [39,40,41,42]. A related architecture, called compact TALENs (or cTALENs), was created by fusing the I-TevI nuclease domain to the C-terminus of TALEs [43]. In each case, the DNA-binding module specifies targeting, simplifying targeting, and engineering requirements, because only a single fusion need sbe constructed to target any site. For the nucleases where I-TevI is fused to the N-terminus of the DNA-binding domain, the reagents have bipartite recognition sites consisting of an I-TevI cleavage motif (5′-CNNNG-3′) that is spaced appropriately from the DNA-binding site. The I-TevI linker domain has some sequence preference for the DNA spacer region that separates the cleavage motif from DNA-binding site [42]. Thus, potential target sites must have both cleavage motif and DNA spacer requirements upstream (5′) of the DNA-binding site. The mechanism of how the I-TevI the domain functions as a monomer to introduce a DSB is not well understood, but it likely involves the rotation of the domain and/or substrate to reposition the domain between nicking reactions [44].

### 3.2. Single-Chain Monomeric Endonucleases

#### 3.2.1. Meganucleases and MegaTALs

Meganucleases (LAGLIDADG homing endonucleases) are naturally found in two forms, dimers or single-chain monomers [8,9]. The class-defining LAGLIDADG motif is found in a α-helix that forms the interface between dimers, or between the N- and C-terminal domains of single-chain monomers. The LAGLIDADG helices also position the acidic residues (the D in LAGLIDADG) near the scissile phosphates. Each meganuclease can tolerate some nucleotide variation in their 12–22 bp native target sites because the enzymes only directly readout a subset of bases throughout their target sites, and because indirect readout plays a role in recognition [45,46]. Indirect readout is also thought to regulate catalytic activity in the central four bases of the target site where the enzyme cleaves DNA. Meganucleases are highly active in cells, with very low or undetectable levels of off-target cleavage, and introduce a 4-nucleotide 3′ overhang upon cleavage that promotes homology-dependent repair (HDR). Re-targeting meganucleases to different sites is labor intensive, requiring engineering of the protein-DNA interface through directed evolution of modules of DNA-contacting residues [47,48,49]. Many re-engineered variants exhibit reduced activity on the target site and require subsequent rounds of further optimization because LHEs are generally intolerant to nucleotide substitutions in the central four bases of their target sites where the enzymes introduce a DSB. Current protocols encourage the selection of target sites with central four sequences identical to the native site. Nonetheless, a number of meganucleases have been successfully targeted to therapeutically relevant sites in the human genome [45,50,51,52,53,54]. Fusion of a TALE domain to meganucleases (called megaTALs) overcomes some issues in meganuclease engineering by providing a programmable, second DNA-targeting domain [55].

#### 3.2.2. CRISPR/Cas9

The Cas9 (CRISPR-associated) protein is a component of the clustered regularly interspersed palindromic repeat (CRISPR) bacterial adaptive immune system. Readers are referred to any number of reviews on the biology, function, and applications of CRISPR/Cas9 [1,2,3]. In terms of genome editing, the most widely used Cas9 protein is from *Streptococcus pyogenes* (Sp), but other Cas9 orthologs have also been investigated [2,3]. SpCas9 is functionally a monomer, with two distinct and unrelated active nuclease sites (RuvC and HNH) generating a blunt-end DSB in the target substrate.

## 4. Regulating Nuclease Activity

Limiting unwanted DSBs or nicks at so-called off-target sites is a primary concern for genome-editing applications as off-target cleavages can lead to chromosome translocations [56,57], cellular toxicity and unforeseen downstream consequences, especially for applications with therapeutic potential. These concerns have become more relevant given recent and high publicity genome-editing studies in human embryos, and approvals for CRISPR/Cas9-based gene therapy trials [13]. A number of detailed studies to capture and identify off-target DSBs by deep sequencing have mapped off-target cleavages for Cas9 and Cas9 variants in tissue culture systems that are reviewed in [58]. Interestingly, a proportion of off-target sites identified by these studies were not predicted by computational methods, suggesting that the prediction algorithms are either based on an incomplete understanding of Cas9-substrate interactions, or Cas9 activity in vivo is influenced by factors that are difficult to predict from in vitro studies (chromatin context, epigenetic modifications, expression levels). Other genome-editing nucleases, including ZFNs, TALENs, and meganucleases have also been profiled for off-target effects [55,59,60,61,62], with TALENs and meganucleases possessing fewer off-target cleavages than ZFNs. It is important to note that direct comparisons of specificity between genome-editing nucleases are extremely difficult, if not impossible, due to the differences in expression levels, protein stability and half-life, binding affinity and mechanism of DNA interaction, and the simple fact that different nucleases will have different off-target sites (Table 2).

Two general strategies that are used to combat issues related to off-target cleavage and cellular toxicity are rationale protein engineering and directed evolution studies of nucleases directed by crystallographic studies, and the utilization of alternative binding or cleavage domains to enhance binding or cleavage specificity. Among the first engineered nucleases with reduced toxicity were ZFNs with FokI nuclease domain variants that were engineered to function only as obligate heterodimers, reducing homodimerization and DNA cleavage between on- and off-target ZFNs [63]. Profiling of wild-type FokI relative to the obligate heterodimers revealed less cellular toxicity and lower levels of off-target cleavage at selected sites. With regard to Cas9, higher fidelity versions of Cas9 (eSpCas9, SpCas9-HF1, and HypaCas9) show substantial reductions in off-target cleavage [64,65,66]. An alternative strategy to reducing off-specific cleavage took advantage of the fact that Cas9 has two nuclease active sites, and mutational inactivation of either the RuvC or HNH sites created a Cas9 nickase [67]. The use of nickase versions rather than nucleases that introduce DSBs had previously been shown for meganucleases, ZF nickases and TALE nickases to reduce toxicity while still promoting genome editing events [68,69,70]. 

Alternative nuclease domains have been used in conjugation with a number of DNA-binding platforms to enhance specificity, including the I-TevI and PvuII domains [33,39]. In the case of the monomeric I-TevI domain, any reduction in the loss of combinatorial specificity resulting from monomeric versus dimeric function is likely compensated by the requirement for and appropriate spacing of the I-TevI cleavage motif (5′-CNNNG-3′) from the binding site. Other strategies to increase specificity of FokI nucleases included swapping the ZF domain for an inactive meganuclease (a LAGLIDADG homing endonuclease) with a longer binding site, and a general intolerance to substitutions [33]. Similarly, the ZF domain of ZFNs was replaced by catalytically inactive versions of Cas9 (dCas9), creating a dimeric RNA-guided FokI with reported increases in specificity relative to Cas9 [34,35,36]. The dCas9-FokI enzymes require the expression of two guide RNAs to appropriately position the FokI nuclease domains for cleavage. To avoid the dimeric requirement of dCas9-FokI fusions, Cas9-ZF fusions were created [32]. These fusions require only a single guide RNA, and interestingly overcome the restriction of the wild-type NGG PAM motif that is required for Cas9 target interaction and R-loop formation, effectively broadening the targeting range.

A number of interesting strategies to regulate Cas9 activity in cells have taken advantage of the modular nature of SpCas9 by splitting the enzyme into domains that are subsequently post-translationally assembled by a self-splicing intein that can be chemically controlled. Similar approaches include rapamycin-induced dimerization [71], optogenetic control of Cas9 assembly by light-sensitive dimerization domains [72], and doxycycline-regulated inducible expression [73], reviewed in [74]. The use of Cas9 inhibitors to control Cas9 activity is an innovative strategy that takes advantage of the molecular arms race between bacterial CRISPR systems and anti-CRISPR proteins that are found encoded in many bacteriophage genomes (a natural target of CRISPR defense) [75,76,77,78]. The molecular details of how anti-CRISPR proteins inhibit Cas9 activity point to a broad range of options for regulating Cas9 activity during genome-editing applications [79]. 

## 5. Biasing Genome-Editing Events

The two most common applications for genome-editing nucleases are to create a knockout, or to knockin (insert) sequence into the genome. These two outcomes rely on different DNA recombination pathways: gene knockouts are predominantly created by the non-homologous end-joining pathway (NHEJ), whereas gene knockins rely on homology directed repair (HDR) that collectively refers to the double-strand break repair, synthesis-dependent strand annealing, and break-induced repair pathways. However, the microhomology-mediated end joining (MMEJ) has been used as an alternative for knockins [80].

**Figure 1:**
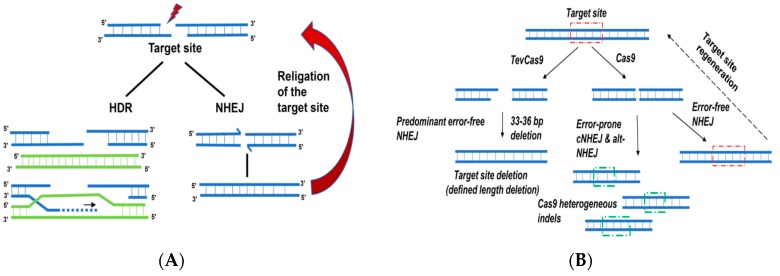
DNA double-strand break repair pathways. (**A**) Homology dependent repair, applicable in the presence of a homologous repair template (green) and error-free non-homologous end joining pathway. The later, however, is responsible for the regeneration of the target site through religation of the paired end complex. Red lightning symbol indicates introduction of a DSB. Large red arrow highlights efficient repair and regeneration of the nuclease target site by NHEJ. (**B**) Schematic model representing how TevCas9 can bias DNA repair outcome (see text for details). Red dashed rectangles, endonuclease target site; green dashed rectangles, mutated target site after error-prone repair. NHEJ, non-homologous end joining; HDR, homology directed repair; cNHEJ, classical NHEJ; alt-NHEJ, alternative-NHEJ.

While all of the genome-editing nucleases have been used to generate gene knockouts, the efficiency of knockouts is influenced by factors such as transfection efficiency of the cell type, the chromatin context of the target site [81,82,83,84], cell cycle stage [85,86], and the type of DSB that is introduced by the nuclease [87,88]. In many mammalian tissue culture systems, nuclease-induced DSBs are substrates for the predominant classical NHEJ pathway (c-NHEJ) that faithfully repairs DSBs in the absence of a repair template to regenerate the target site for re-cleavage by the nuclease, potentially creating a persistent cycle of cleavage and ligation that is not productive for genome editing (Figure 1A). The blunt DNA ends generated by Cas9 are preferred substrates for this type of repair. This persistent cycle is perturbed when the DSB is repaired by error-prone NHEJ pathways, resulting in small insertions or deletions (indels) at the break site [89]. Sequencing of modified alleles from populations of edited cells revealed a spectrum of indel lengths that resulted from individual repair events at each DSB, some of which potentially change the reading frame of the targeted gene leading to a non-functional protein (a knockout) [42]. Thus, while many gene-editing studies report on the overall efficiency of modification from a population of modified cells, often by mismatch cleavage assays [90,91,92], downstream identification and the isolation of defined knockouts is complicated the heterogeneous nature of indels, requiring the screening of individual clonal lines. One strategy to bias NHEJ events towards deletions of defined lengths involved the use of a dual-active site nuclease created by the fusion of the nuclease domain of I-TevI to a meganuclease (MegaTev) or to Cas9 (TevCas9). In either case, the MegaTev or TevCas9 introduces two DSBs at a target site, one at the I-TevI cleavage site, and the other at the meganuclease of Cas9 cleavage sites. This dual DSB effectively deletes the intervening sequence between the two cut sites, with two consequences. First, the target site for the nuclease is deleted after the NHEJ repair, and thus is not a substrate for re-cleavage. Second, the majority of events at the target site generate a defined-length deletion, with the length being determined by the distance between the I-TevI and meganuclease or Cas9 cut sites, further reducing the complexity of downstream screening for defined gene knockouts (Figure 1B).

In contrast to the ease of generating gene knockouts, the rates of nuclease-induced HDR can vary considerably. In cultured mammalian cells, rates of HDR are very low, and efforts to downregulate NHEJ by use of chemical inhibitor or transient knockdown of NHEJ proteins to enhance HDR have been met with variable success. For example, the sequestration of the DNA ligase IV protein by SCR7 resulted in increased HDR efficiency in cell lines and mice models [93]. In another approach, the rational design of single-stranded DNA (ssDNA) donors (asymmetric donor DNA) enhanced Cas9-induced HDR events up to ~60% [89]. However, rates of HDR in primary cell lines or in vivo are significantly lower than in cultured cell lines, and are further complicated by the need for co-delivery of a repair template in addition to the nuclease. A major stumbling block to increasing HDR efficiency has been the lack of simple and robust method to measure HDR and NHEJ directly at the target site [94]. The first attempt to do so was the traffic light reporter system that reported on HDR or NHEJ events by reading frame shifts that were produced in fluorescent reporter genes when repaired after a nuclease-introduced DSB [95]. More recently, a digital, droplet PCR-based assay has been developed that can simultaneously detect one HDR or NHEJ event out of 1000 copies of the genome [94]. In addition, high resolution melting analysis (HRMA) for the rapid identification of efficient editing can distinguish between the melting profiles of PCR products amplified from the modified target site relative to an unmodified site [96].

It is generally accepted that HDR pathways are most efficient when presented with a 3′ overhang at DSBs. Thus, one contributing factor to HDR efficiency with genome-editing nucleases is the type of DNA ends that are introduced. Comparison of HDR versus NHEJ efficiency with ZFNs, megaTALs, and TALENs indicated that the 3′ overhangs that were generated by megaTALs promoted high rates of HDR [50], where as some studies have suggested high rates of HDR using ZFNs [97]. Gene knockins with Cas9 suffer from the fact that the blunt DNA ends produced by Cas9 are poor substrates for HDR pathways [98]. The use of paired Cas9 nickases that introduce two staggered nicks to generate a DSB with a 5′ overhang can increase HDR [67], as can use of asymmetric single-stranded oligonucleotide substrates (ssODNs) [89]. The Cpf1 (CRISPR from *Prevotella* and *Francisella*) nuclease, derived from a type I CRISPR system, generates a 5 nucleotide 5′ overhang at cleavage, and may be better suited for HDR applications than Cas9 [99]. Manipulating Cas9 and other nucleases to generate DNA ends more favorably for HDR is an active area of research [100], and could be accomplished by using the dual TevCas9 nuclease that generates a mixed DNA end (a 3′ I-TevI break and a blunt end Cas9 break). Alternatively, a TevCas9 variant with a Cas9 H840A substitution to inactivate the HNH active site would generate a cleavage product with a ~38-nt single-strand 3′ overhang, conceivably biasing events towards HDR. 

## 6. Conclusions

The history of the development of genome-editing nucleases has been characterized by bursts of enthusiasm and widespread adoption of new reagents over existing ones, followed by realization of limitations and subsequent reagent optimization. Is there a need for alternative genome-editing reagents in the age of CRISPR/Cas9? We would argue yes, simply because Cas9 is not suitable for all types of genome-editing applications. For rapid gene knockouts in cultured cell lines or model organisms, Cas9 and related proteins are the obvious choice. Optimizing Cas9 HDR applications is an active area of investigation. However, Cas9 is more than just a nuclease, as it has become a platform for targeting other types of editing activities, notably base deaminases [101,102,103,104] and methyltransferases [105,106,107]. In this regard, the fusion of alternative nucleases (such as I-TevI) to Cas9 could generate reagents with novel activities and applications that would expand the Cas9 genome-editing toolbox.

## Figures and Tables

**Table 1:** Genome-editing nucleases.

Nuclease Domain	Property	Type of DSB	Associated Reagents
FokI	Type IIS, dimeric, non-specific nuclease	3-nt 5′ overhang	ZFNs, TALENs, Cas9-FokI
I-TevI	GIY-YIG, monomeric, site-specific	2-nt 3′ overhang	ZFEs, TALENs, MegaTev, TevCas9
PvuII	Type IIP, homodimeric, site-specific	Blunt end	I-SceI-PvuII, ZF-PvuII, PvuII-LHE
Recombinase	Serine recombinase (Sin recombinase); invertase Gin	Not applicable	ZF-recombinase, TALE-recombinase
Cas9	Type II CRISPR/Cas family; RuvC/HNH nuclease domains; monomeric; requires PAM sequence; moderate specificity	Blunt end	CRISPR/Cas9, CRISPRi, Cas9-FokI
Cpf1	Monomeric; non-specific; recognizes T-rich PAM sequence at the 5′ of the guide RNA	5-nt 5′ overhang	CRISPR/Cpf1
Meganuclease	LAGLIDADG family, monomeric or dimeric; very specific	4-nt 3′ overhang	MegaTAL, TALE-I-SceI, MegaTev

DSB, double-strand break; ZFNs, zinc-finger nucleases; Cas9, CRISPR-associated protein 9; GIY-YIG, the GIY-YIG family of homing endonucleases; MegaTev, a chimeric fusion of a meganuclease and the catalytic and linker domains of I-TevI; ZF, zinc finger; LHE, LAGLIDADG homing endonuclease; CRISPR, clustered regularly interspaced short palindromic repeats; PAM, protospacer-associated motif; Sin, Staphylococcal invertase-like gene; Gin, G-segment invertase; Cpf1, CRISPR from *Prevotella* and *Francisella*; RuvC, ultraviolet-light sensitive gene C; HNH, His-Me finger nuclease domain.

**Table 2:** Comparison of four commonly used genome-editing nucleases.

Property	Cas9	ZFN	TALEN	Meganuclease
Specificity (off-target)	Relatively non-specific	Relatively non-specific	Specific	Very specific
Biasing events (repair)	NHEJ	NHEJ	HDR	HDR
Design & targeting constraints	PAM requirement (NGG for SpCas9)	Context-dependent assembly of ZFs; GC rich targets preferred	Assembly of TALE repeats; 5′ targeted base is T	Re-design of protein-DNA interface; central 4 bases intolerant to change
Dimerization required	No	Yes	Yes	No
Coding sequence	Long	Short	Long and repetitive	Short
Therapeutic delivery	Easy	Moderate	Moderate	Easy
Vector packaging	Moderate	Difficult	Difficult	Easy
Multiplex potential	High	Low	Low	High
Cost-effective	Yes	No	Moderate	No

NHEJ, non-homologous end joining; HDR, homology-directed repair.
